# Draft Genome Sequence of Streptococcus thermophilus Strain CBC-S77, Isolated from Homemade Dairy Foods in Bulgaria

**DOI:** 10.1128/MRA.00879-20

**Published:** 2020-09-10

**Authors:** Hanan R. Shehata, Richmond A. Chandler, Steven G. Newmaster

**Affiliations:** a NHP Research Alliance, College of Biological Sciences, University of Guelph, Guelph, Ontario, Canada; b Department of Microbiology, Faculty of Pharmacy, Mansoura University, Mansoura, Egypt; c Chandler Biopharmaceutical Corp., Milton, Ontario, Canada; University of Maryland School of Medicine

## Abstract

Here, we report the draft genome sequence of Streptococcus thermophilus strain CBC-S77. The strain was originally isolated from naturally processed, homemade dairy foods in West Rhode Mountain, Bulgaria. The genome was assembled in 148 contigs with a total length of 1,707,130 bp, with 1,563 coding genes and a GC content of 39.11%.

## ANNOUNCEMENT

Streptococcus thermophilus is a species of lactic acid bacteria that is commonly used as a starter culture in the dairy industry, especially for yogurt and cheese (1–3). In addition to its role in milk fermentation, it plays an important role in enhancing the flavor and texture of yogurt (4–6). Streptococcus thermophilus was also reported to have various health benefits, such as antimicrobial activity (7), anti-inflammatory potential (8), a role in the alleviation of lactose intolerance (3), gastroprotective effects (9), and antioxidant and immunomodulation activities (3).

Streptococcus thermophilus strain CBC-S77 was isolated from naturally processed, homemade dairy foods from an ecologically pure and chemically unaffected geographical area (West Rhode Mountain, Bulgaria) in May 1977. Streptococcus thermophilus strain CBC-S77 was isolated on M17 agar (Oxoid) plates that were incubated anaerobically at 42°C for 72 h (10). Here, we present the genome sequence of Streptococcus thermophilus strain CBC-S77. The purpose of sequencing the genome of strain CBC-S77 is to better understand the probiotic properties and the underlying mechanisms of this strain and to confirm its safety for human consumption.

Strain CBC-S77 was obtained from Chandler Biopharmaceutical Corp. as a lyophilized powder. The NucleoSpin food kit (product number 740945.50; Macherey-Nagel, Germany) was used for genomic DNA extraction directly from 50 mg of the lyophilized powder. The extraction was performed according to the manufacturer’s instructions. A Qubit v4.0 fluorometer was used for DNA quantification, and the DNA was diluted to 2 ng/μl. The DNA was then submitted to the Advanced Analysis Centre Genomics Facility at the University of Guelph (Guelph, ON, Canada) for library preparation and sequencing on an Illumina MiSeq system using an Illumina Nextera XT kit and Illumina MiSeq reagent v3 kit (600 cycles; 2 × 300-bp reads).

CLC Genomics Workbench v20.0.3 (Qiagen Bioinformatics) was used to analyze the sequencing output. Default parameters were used except where otherwise noted. First, the reads were subjected to quality trimming, in which low-quality sequences (limit of 0.05 base-calling error probability) were removed and a maximum of 2 ambiguous nucleotides were allowed. The total numbers of paired reads before and after quality trimming were 2,285,426 and 2,279,381 reads, respectively. High-quality reads with a total of 331,600,799 bases were used in *de novo* assembly, yielding ∼190× coverage. The genome was assembled in 148 contigs, with a total length of 1,707,130 bp, a GC content of 39.11%, and an *N*_50_ value of 9,389 bp. The ContEst16S tool (11) was used to extract the full-length 16S rRNA gene sequence from the genome sequence, and the gene sequence was used for a BLAST search with GenBank to confirm species identity (12). The NCBI Prokaryotic Genome Annotation Pipeline (PGAP) v4.11 (https://www.ncbi.nlm.nih.gov/genome/annotation_prok) was used for genome annotation, which predicted 1,563 protein-coding sequences and 39 tRNA genes.

### Data availability.

This whole-genome shotgun project has been deposited in DDBJ/ENA/GenBank under the accession number JABWOT000000000. The version described in this paper is version JABWOT000000000.1. The raw files were deposited in the SRA database under accession number SRR12037890.
